# Bipolarized intrinsic faradaic layer on a semiconductor surface under illumination

**DOI:** 10.1093/nsr/nwac249

**Published:** 2022-11-04

**Authors:** Mengfan Xue, Zhiqiang Chu, Dongjian Jiang, Hongzheng Dong, Pin Wang, Gengzhi Sun, Yingfang Yao, Wenjun Luo, Zhigang Zou

**Affiliations:** Eco-Materials and Renewable Energy Research Center (ERERC), Jiangsu Key Laboratory for Nano Technology, National Laboratory of Solid State Microstructures and Department of Physics, Nanjing University, Nanjing 210093, China; Eco-Materials and Renewable Energy Research Center (ERERC), Jiangsu Key Laboratory for Nano Technology, National Laboratory of Solid State Microstructures and Department of Physics, Nanjing University, Nanjing 210093, China; National Laboratory of Solid State Microstructures, College of Engineering and Applied Sciences, Nanjing University, Nanjing 210093, China; National Laboratory of Solid State Microstructures, College of Engineering and Applied Sciences, Nanjing University, Nanjing 210093, China; Eco-Materials and Renewable Energy Research Center (ERERC), Jiangsu Key Laboratory for Nano Technology, National Laboratory of Solid State Microstructures and Department of Physics, Nanjing University, Nanjing 210093, China; Key Laboratory of Flexible Electronics (KLOFE) and Institute of Advanced Materials (IAM), Nanjing Tech University, Nanjing 211816, China; National Laboratory of Solid State Microstructures, College of Engineering and Applied Sciences, Nanjing University, Nanjing 210093, China; National Laboratory of Solid State Microstructures, College of Engineering and Applied Sciences, Nanjing University, Nanjing 210093, China; Eco-Materials and Renewable Energy Research Center (ERERC), Jiangsu Key Laboratory for Nano Technology, National Laboratory of Solid State Microstructures and Department of Physics, Nanjing University, Nanjing 210093, China; National Laboratory of Solid State Microstructures, College of Engineering and Applied Sciences, Nanjing University, Nanjing 210093, China

**Keywords:** semiconductor surface, interface charge transfer, reduction faradaic layer, oxidation faradaic layer, faradaic layer descriptor

## Abstract

Interface charge transfer plays a key role in the performance of semiconductors for different kinds of solar energy utilization, such as photocatalysis, photoelectrocatalysis, photochromism and photo-induced superhydrophilicity. In previous studies, different mechanisms have been used to understand interface charge transfer processes. However, the charge transfer mechanism at the solid/liquid interface remains a controversial topic. Here, taking TiO_2_ as a model, we find and prove, via experiments, the new characteristic of photo-induced bipolarity of the surface layer (reduction faradaic layer and oxidation faradaic layer) on a semiconductor for the first time. Different from energy level positions in the classic surface states transfer mechanism, the potential window of a surface faradaic layer is located out of the forbidden band. Moreover, we find that the reduction faradaic layer and oxidation faradaic layer serve as electron and hole transfer mediators in photocatalysis, while the bipolarity or mono-polarity of the surface layer on a semiconductor depends on the applied potential in photoelectrocatalysis. The new characteristic of bipolarity can also offer new insights into the charge transfer process at the semiconductor/liquid interface for solar energy utilization.

## Teaser

Luo et al. find the photo-induced bipolarity characteristic of a faradaic layer on semiconductor surface and also propose electrochemical potential as a simplified descriptor for the faradaic layer.

## INTRODUCTION

In the past few decades, different semiconductors have been widely used as light absorbers in photocatalysis [1,2], photoelectrocatalysis [3,4], photochromism [5,6] and photo-induced superhydrophilicity [7,8]. When semiconductors are illuminated, photo-generated electrons and holes in the bulk transfer to the surface and react with adsorbed species. Interface charge transfer plays a key role in the performance of a semiconductor. Three kinds of mechanisms—direct transfer, surface states transfer and intrinsic faradaic layer transfer—have been proposed in previous studies (Fig. S1). In the direct transfer model, photo-generated electrons in the conduction band and holes in the valence band transfer to the redox in the electrolyte directly [9,10]. However, according to Marcus theory, interface charge transfer should take place when the electron energy in the electrodes equals that of the redox couples in the electrolyte. For those semiconductors with a wide band gap, such as TiO_2_, a low photocurrent is predicted due to small overlapping between the energy band and energy states of a redox couple. However, a high photocurrent and incident photon-to-electron conversion efficiency (IPCE) can be observed in TiO_2_ in experiments, which suggests that the direct transfer mechanism is not suitable for explaining the interface charge transfer process [11–13]. Therefore, a surface state transfer model is proposed, in which photo-generated carriers are firstly trapped in surface states, and then transfer to the redox. The surface states include physical states (dangling bonds or defects) and chemical states (adsorbed species), which lead to discreet energy levels within the forbidden band [13]. The energy level positions of surface states have significant effects on interface charge transfer. To date, the roles of the surface states are still under debate. Some studies suggest that surface states play a role as recombination centers and decrease performance [14–16], but others think surface states serve as interface charge transfer mediators for redox reactions [17–21]. These inconsistent results on the surface states hinder clear understanding of interface charge transfer in a semiconductor. Another mechanism of the intrinsic faradaic layer is also proposed to understand interface charge transfer, in which there is an intermediate layer with coupled electron and ion transfer at the solid/liquid interface [22]. However, no direct experimental evidence on the intrinsic faradaic layer is given in the previous study. Moreover, the intrinsic faradaic layer indicates mono-polarity on the semiconductor surface, which cannot explain why oxidation and reduction reactions simultaneously happen on the surface of a photocatalyst. Therefore, it is important to clarify the composition, the energy level positions and the characteristics of the semiconductor surface via experiments.

In this work, using a TiO_2_ semiconductor as a model, we find a new characteristic of photo-induced bipolarity of the intrinsic faradaic layer on the semiconductor surface. We investigated the surface composition and the potential window of the intrinsic faradaic layer by *in situ* X-ray photoelectron spectroscopy (XPS), *in situ* electron paramagnetic resonance (EPR), time-of-flight secondary-ion mass spectroscopy (TOF-SIMS) and electrochemical methods. A reduction faradaic layer (RFL) and an oxidation faradaic layer (OFL) are obtained under illumination simultaneously. We find the potential window of the RFL is located out of the forbidden band, which is contrary to conventional energy level positions of surface states in semiconductors. Moreover, the roles of the RFL and OFL in photocatalysis and photoelectrocatalysis were investigated. In photocatalysis, the RFL and OFL serve as electron and hole transfer mediators, while the bipolarity or mono-polarity of the intrinsic faradaic layer on a semiconductor depends on the applied potential in photoelectrocatalysis. These results can also be used to understand the photochemical process in photochromism and photo-induced superhydrophilicity, and to provide a unified understanding that describes charge transfer at the semiconductor/liquid interface.

## RESULTS AND DISCUSSION

**Figure 1.**
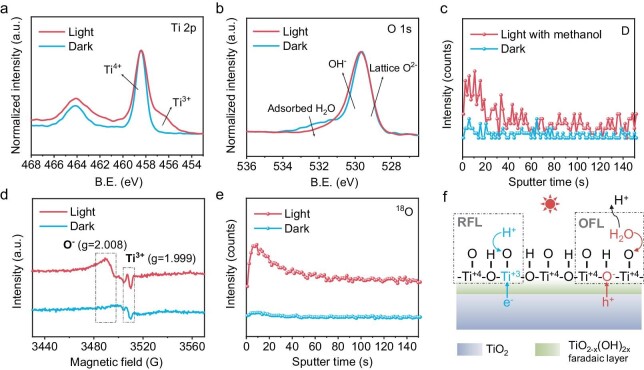
*In situ* XPS spectra of (a) Ti 2p and (b) O 1s in TiO_2_ in the dark and under illumination; (c) TOF-SIMS spectra of D depth profile of TiO_2_ in the dark and under illumination, in a mixture solution of D_2_O and H_2_^18^O with and without methanol as hole scavenger; (d) *in situ* EPR spectra of TiO_2_ in the dark and under illumination in N_2_; (e) TOF-SIMS spectra of ^18^O depth profile of TiO_2_ in the dark and under illumination, in a mixture solution of D_2_O and H_2_^18^O; (f) schematic diagram for the surface composition of TiO_2_ under illumination.

A TiO_2_ film was deposited on an SnO_2_:F (FTO) substrate by the conventional hydrothermal method and the preparation details are shown in the supplementary information [23]. The as-deposited TiO_2_ is rutile (Fig. S2). *In situ* XPS, *in situ* EPR and TOF-SIMS were used to detect surface composition on TiO_2_ under illumination. Only XPS peaks of Ti^4+^ at 458.3 eV and 464.0 eV are observed on the TiO_2_ surface in the dark, while new peaks at 456.6 eV and 462.6 eV occur under illumination, and are attributed to Ti^3+^ (Fig. 1a) [24]. The results suggest that Ti^4+^ on the surface is reduced into Ti^3+^ under illumination. Moreover, the content of adsorbed H_2_O on the TiO_2_ surface remarkably decreases under illumination, which suggests that adsorbed H_2_O is consumed and participates in photo-induced surface reactions. In order to further confirm the photo-induced reactions between H_2_O and the surface of TiO_2_, we conducted isotope labeling experiments by immersing TiO_2_ in a mixture solution of H_2_^18^O and D_2_O with and without methanol as hole scavenger in the dark and under illumination. A surface reduction reaction of Ti^4+^ into Ti^3+^ requires the participation of H^+^ to maintain electric neutrality. TOF-SIMS was used to detect D depth profile in TiO_2_ and the results are shown in Fig. 1c. No signal of D is observed on the surface of TiO_2_ in the dark. Under illumination, D is observed on the surface of TiO_2_ in the mixture solution of H_2_^18^O and D_2_O with methanol, which suggests that a proton indeed reacts with the surface of TiO_2_ under illumination. However, we do not observe D on TiO_2_ in the mixture solution without methanol under illumination (Fig. S3). This is because the isotope labeling experiments are *ex situ* and the photo-reduced Ti^3+^ on the surface is easily re-oxidized by oxygen in the air. The methanol as hole scavenger can make D diffuse deeper into the surface of TiO_2_. Therefore, a photo-induced reduction reaction is proposed as Ti^+4^O_2−x_(OH)_2x_ + H^+^ + e^−^ ↔ Ti^+3^O_1−x_(OH) _2x+1_, which is a faradaic reaction with coupled electron and ion transfer.

In Fig. 1a–c, only a surface reduction reaction, but no oxidation reaction, can be observed. *In situ* EPR was used to detect oxidation intermediates. Under illumination, a stronger signal at g = 2.008 is observed compared with the sample in the dark (Fig. 1d), which is ascribed to O^−^ due to the oxidation of O^2−^ by photo-generated holes in TiO_2_ [25]. Moreover, a signal of Ti^3+^ at g = 1.999 is also observed both in the dark and under illumination. The Ti^3+^ in the dark possibly comes from oxygen defects in the as-deposited TiO_2_ [26]. Different from *in situ* XPS, the oxidation reaction can be observed by EPR. This is because XPS can only detect the surface composition, but EPR can detect the composition both on the surface and in the bulk [27]. The surface O^−^ oxidized by photo-generated holes can be re-reduced into O^2−^ by adsorbed H_2_O. To confirm the photo-induced oxidation reaction between H_2_O and the surface of TiO_2_, the ^18^O depth profile is shown in Fig. 1e. No obvious signal of ^18^O is observed on TiO_2_ in the dark. Therefore, the adsorption of H_2_^18^O on the surface of TiO_2_ is negligible. Under illumination, the signal of ^18^O increases remarkably. The results suggest that H_2_O indeed reacts with the surface of TiO_2_ under illumination. The ^18^O distributes uniformly in TiO_2_ at a greater depth, which is not because the ^18^O can diffuse into such depths but due to the loose morphology of TiO_2_ (Fig. S4). Therefore, isotope labeling experiments were also carried out on a single crystal rutile TiO_2_ (110) and the results are shown in Fig. S5. Different from the TiO_2_ film, the signal of ^18^O gradually decreases with an increase in depth of the single crystal TiO_2_ (110) under illumination, and most of the ^18^O is distributed on the surface of TiO_2_. According to the above results and analysis, we propose a surface oxidation faradaic reaction as Ti^+4^O_2−x_(OH)_2x_ + H_2_O + h^+^ ↔ Ti^+4^O^−1^O_1−x_(OH) _2x+1_ + H^+^. The composition on the surface of TiO_2_ in the dark is shown in Fig. S6. It is clear that both D and ^18^O participate in the photo-induced redox reactions and produce a new composition on the surface of TiO_2_. Thus, photo-induced surface reactions include a reduction faradaic reaction and oxidation faradaic reaction, which are bipolarized and called the RFL and OFL, respectively. Moreover, the RFL and OFL are blocked by the neutral Ti^+4^O_2−x_ (OH)_2x_ (Fig. 1f).

**Figure 2.**
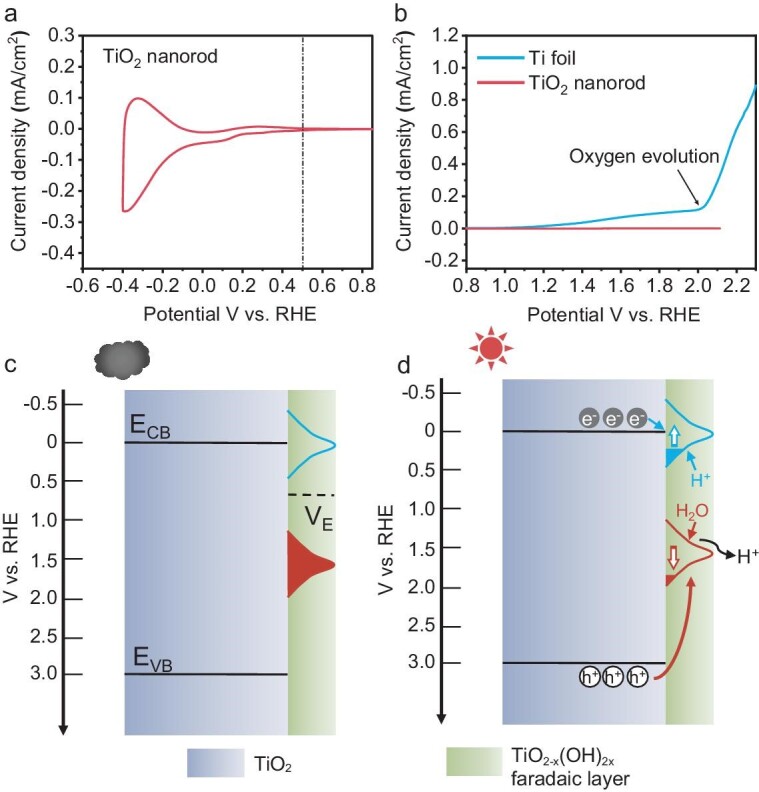
(a) CV curve of TiO_2_ in the dark and (b) LSV curves of Ti foil and TiO_2_ in the dark, 1 M phosphate buffer solution (pH∼7), 20 mV/s; schematic diagrams for energy band positions and potential windows of the RFL and OFL for TiO_2_ (c) in the dark and (d) under illumination; E_VB_ and E_CB_ are the valence band and conduction band of a semiconductor, respectively; V_E_ is the equilibrium potential.

In order to investigate the potential windows of the photo-induced RFL and OFL, cyclic voltammetry (CV) curves of TiO_2_ and Ti foil were measured and the results are shown in Fig. 2a and b. Electro-induced insertion of D into TiO_2_ is also observed in the dark (Fig. S7), which is similar to the photo-induced reduction reaction [28]. Therefore, electrochemical methods can be used to simulate the half reactions in the photochemical process. The CV curve of TiO_2_ suggests that the potential window of the RFL is −0.4∼0.5 V_RHE_ (Fig. 2a) [29]. However, no potential window of OFL is observed in the dark even at a very high potential of 2 V_RHE_ (Fig. 2b). Similar results are also observed on single-crystal TiO_2_ (110) (Fig. S8). This is because TiO_2_ has high resistance, which leads to most of the voltage drop in the bulk. The surface voltage is negligible. Therefore, the surface of TiO_2_ cannot be oxidized in the dark. In order to measure the potential window of OFL, Ti foil was introduced for electrochemical measurement due to its significantly lower bulk resistance but similar surface composition, TiO_2−x_(OH)_2x_, to that of TiO_2_ (Fig. S9). A linear sweep voltammetry (LSV) curve of Ti foil shows that an oxidation current appears at around 1.2 V_RHE_ and an oxygen evolution reaction happens at a potential higher than 2 V_RHE_, where bubbles are observed (Fig. 2b) [30]. Therefore, the potential window of OFL is ∼1.2∼2.0 V_RHE_. Moreover, the energy band positions of TiO_2_ were also measured by UV-Vis spectra and the Mott-Schottky method (Fig. S10). According to these results, the energy band positions and the potential windows of the RFL and OFL of TiO_2_ are plotted in Fig. 2c and d. Moreover, the TiO_2_ indicates an open-circuit potential of ∼0.6 V_RHE_ in the dark (Fig. S11), which is an equilibrium potential (V_E_) between TiO_2_ and electrolyte. Since the equilibrium potential lies between the potential windows of the RFL and OFL, no surface reduction or oxidation reaction happens and only Ti^4+^ exists on the surface in the dark (Fig. S5). The conduction band position of TiO_2_ is located within the potential window of the RFL. Moreover, the valence band position of TiO_2_ is more positive than the potential window of the OFL. When TiO_2_ is illuminated, photo-generated electrons transfer from the conduction band to the RFL, while photo-generated holes transfer from the valence band to the OFL. A similar bipolarized RFL and OFL are also observed on the Fe_2_O_3_ surface (Figs S12–S16 and Table S1). Though we investigated the bipolarity of intrinsic faradaic layers on metal oxide semiconductors in this study, it is possible that the bipolarized intrinsic faradaic layers also exist on other types of semiconductors, such as sulfide and polymer semiconductors.

**Figure 3.**
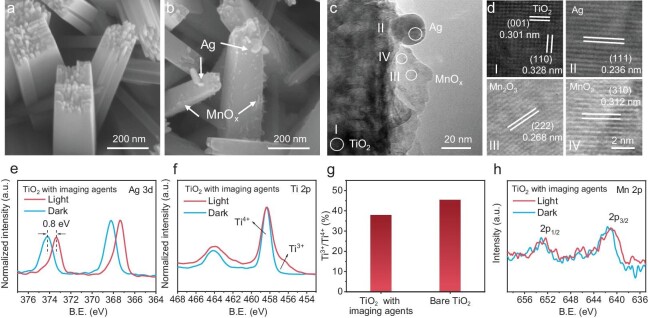
SEM images of TiO_2_ (a) without imaging agents and (b) with imaging agents of Mn^2+^ and Ag^+^; (c) TEM images of TiO_2_ with imaging agents of Mn^2+^ and Ag^+^ and (d) enlarged images in c; *in situ* XPS spectra of (e) Ag 3d and (f) Ti 2p in TiO_2_ with imaging agents of Mn^2+^ and Ag^+^ in the dark and under illumination; (g) the ratio of Ti^3+^/Ti^4+^ in TiO_2_ with imaging agents of Mn^2+^ and Ag^+^ and bare TiO_2_ under illumination; (h) *in situ* XPS spectra of Mn 2p in TiO_2_ with imaging agents of Mn^2+^ and Ag^+^.

In order to investigate the roles of the RFL and OFL during interface charge transfer in photocatalysis, TiO_2_ was illuminated in aqueous solution with Mn^2+^ as hole imaging agent and Ag^+^ as electron imaging agent. The scanning electron microscope (SEM) images of TiO_2_ with and without imaging agents of Mn^2+^ and Ag^+^ are shown in Fig. 3a and b, respectively. Without imaging agents, bare TiO_2_ is composed of nanorod arrays with a smooth surface. After illumination with imaging agents of Mn^2+^ and Ag^+^, flakes and particles are both deposited on the (110) facet of the TiO_2_ nanorod (Fig. 3b). The flakes and particles are identified as Mn_2_O_3_/MnO_2_ (MnO_x_) and metal Ag^0^ by transmission electron microscope (TEM) images in Fig. 3c and d, and by XPS in Fig. S17. Considering the ions of Mn^2+^ and Ag^+^ in the solution, the solid deposited products, MnO_x_ and Ag, on the surface of TiO_2_, are obtained by oxidation and reduction reactions, respectively. The results are different from previous studies, in which electron and hole imaging agents are deposited on different facets of the semiconductor under illumination [31]. In order to further confirm that both MnO_x_ and Ag are deposited on the same facet of the TiO_2_ nanorod, co-deposition of MnO_x_ and Ag were carried out on the (110) facet surface of the TiO_2_ single crystal. Similar to the nanorod sample, both MnO_x_ flakes and Ag particles are also observed on the (110) facet (Fig. S18). Moreover, single deposition of MnO_x_ or Ag was also carried out on the surface of TiO_2_ to exclude the effects of interrelation between Mn^2+^ and Ag^+^ during photo-generated carrier transfer. MnO_x_ flakes or Ag particles distribute on the (110) facet of the TiO_2_ nanorod, although some Ag particles are also observed on the top of the nanorod (Figs S19 and S20). The results suggest that the single deposition and co-deposition indicate the same transfer path of photo-generated carries.

In order to understand the roles of the RFL and OFL in interface charge transfer, *in situ* XPS was also used to characterize TiO_2_ with and without imaging agents of Mn^2+^ and Ag^+^ on the surface, and the results are shown in Figs 1a and 3e–g. Under illumination, the binding energies at 373.4 eV of Ag 3d in TiO_2_, with imaging agents of Mn^2+^ and Ag^+^, shift 0.8 eV negatively from 374.2 eV of the sample in the dark, which suggests that Ag^+^ is reduced into Ag^0^ (Fig. 3e) [31]. At the same time, Ti^3+^ is also observed (Fig. 3f). However, the ratio of Ti^3+^/Ti^4+^ is 38% on the TiO_2_ with imaging agents of Mn^2+^ and Ag^+^, which is lower than the value of 45% in a bare TiO_2_. Some previous studies also suggest that photo-induced reduction products on the semiconductor surface are active sites for photocatalysis [32,33]. Therefore, the decreased content of Ti^3+^ is used to reduce Ag^+^ into Ag^0^. In contrast, no obvious change of the valence state of Mn can be observed under illumination (Fig. 3h). The results are in contrast with aforementioned results where the TEM in Fig. 3d suggests that Mn^2+^ is oxidized to MnO_x_. Considering that the oxidation of Mn^2+^ into MnO_x_ needs participation of ions, the oxidation of Mn^2+^ happens in the solution, but not in a vacuum. Unlike the oxidation of Mn^2+^ into MnO_x_, the reduction of Ag^+^ into Ag^0^ does not need the participation of ions and can be observed by *in situ* XPS. The results suggest that the RFL plays a role as transfer mediator of photo-generated electrons in photocatalysis.

**Figure 4.**
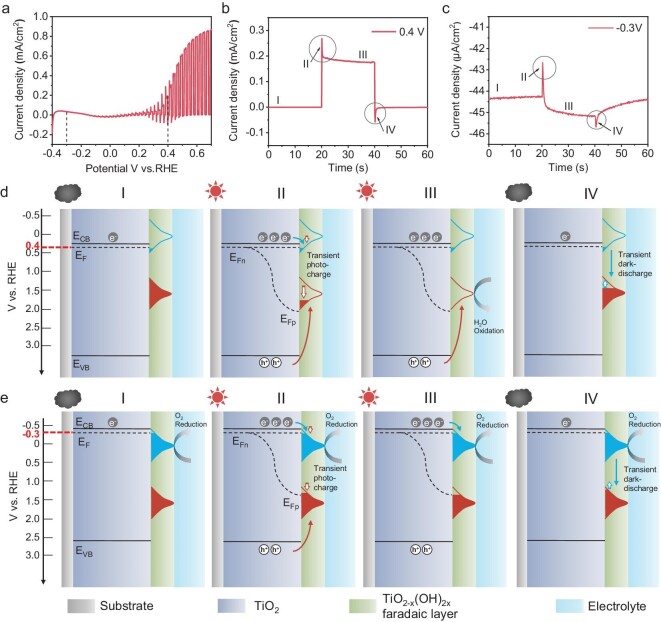
(a) LSV curve of TiO_2_ under chopped illumination in 1 M phosphate buffer solution (pH∼7), 20 mV/s; i-t curves of TiO_2_ at (b) 0.4 V_RHE_ and (c) −0.3 V_RHE_ under chopped illumination; schematic diagrams for energy band positions and potential windows of the RFL and OFL of TiO_2_ for the charge transfer process at (d) 0.4 V_RHE_ and (e) −0.3 V_RHE_ in the dark and under illumination. E_F_ represents the Fermi level of electrons; E_Fn_ and E_Fp_ represent the quasi-Fermi levels of electrons and holes, respectively.

In addition to serving as charge transfer mediator in photocatalysis, the roles of the RFL and OFL in photoelectrocatalysis are also investigated by LSV and i-t curves and the results are shown in Fig. 4a–c, respectively. A potential window of −0.3 V_RHE_ to 0.5 V_RHE_ for a transient photocurrent and a potential window of higher than 0.3 V_RHE_ for a steady photocurrent are observed in TiO_2_ under chopped illumination (Fig. 4a and Fig. S21). In previous studies, the steady photocurrent comes from water oxidation [34]. However, the origin of the transient photocurrent, recombination or trapping of photo-generated carriers on the surface, is still under debate [34,35]. The intrinsic faradaic layer on the semiconductor surface is a crucial part of a solid/liquid faradaic junction, which can be used to understand the transient photocurrent. The photo-generated holes are stored in the OFL under illumination, which is similar to the photo charge process in an extrinsic faradaic junction for solar rechargeable devices [36]. The potential window of the OFL in the dark can shift to the flat potential of a semiconductor under illumination. The transient photocurrent comes from the photo-charge process of the OFL, which decreases to zero when the potential of the OFL is the same as the quasi-hole Fermi level of TiO_2_. In contrast, the steady photocurrent is observed at a potential higher than 0.3 V_RHE_ because the stored holes in the OFL can further transfer to water for oxygen production at such a high potential. Water can be continuously supplied from electrolyte. Therefore, the OFL plays a role as hole transfer mediator at a potential higher than 0.3 V_RHE_.

In order to further understand the roles of the RFL and OFL in photoelectrocatalysis, i-t curves at the potentials of 0.4 V_RHE_ and −0.3 V_RHE_ were also measured and the results are shown in Fig. 4b and c. Accordant transient photocurrents but opposite steady photocurrents are observed at the potentials of 0.4 V_RHE_ and −0.3 V_RHE_, respectively. Stage I, II, III and IV are dark equilibrium, light-on response, photo equilibrium and light-off response, respectively. Figure 4d and e show energy band positions and potential windows of the RFL and OFL of TiO_2_ at the potentials 0.4 V_RHE_ and −0.3 V_RHE_, respectively. The difference between the quasi-Fermi levels of electrons and holes under illumination is derived from Fig. S22. In dark equilibrium at 0.4 V_RHE_ (Stage I, Fig. 4d), the Fermi level of a semiconductor is controlled by the applied potential, which is located within the potential window of the RFL and much more negative than the potential window of the OFL. Therefore, the RFL is filled to 0.4 V_RHE_ with charges (coupled electrons and ions), while the potential window of the OFL is completely filled in the dark. When the light is on, photo-generated holes in TiO_2_ can fill the RFL and OFL (Stage II, Fig. 4d), which leads to the transient photocurrent as mentioned above. After all the OFL is charged by photo-generated holes, the hole quasi-Fermi level in TiO_2_ is more positive than the potential window of the OFL, which leads to water oxidation and a steady current being obtained (Stage III, Fig. 4d). When the light is off, the stored holes in the OFL are released to TiO_2_ through the RFL (Stage IV, Fig. 4d). Moreover, the role of the OFL in the charge transfer process at 0.6 V_RHE_ is also shown in Fig. S23. At a potential of 0.6 V_RHE_, electron quasi-Fermi level is more positive than the potential window of the RFL, the performance of water oxidation only depends on the OFL and the stored holes in the OFL cannot be released into TiO_2_ (Stage IV in Fig. S23). Therefore, the saturated photocurrent is determined by the OFL and no transient light-off response is observed. Moreover, in dark equilibrium at −0.3 V_RHE_ (Stage I, Fig. 4e), the Fermi level of a semiconductor is controlled by the applied potential, and obvious steady cathodic current is observed, which comes from the oxygen reduction reaction (Fig. S24). When the light is on, photo-generated holes in TiO_2_ charge both the RFL and OFL (Stage II, Fig. 4e), which leads to the accordant transient photocurrent at the potential of 0.4 V_RHE_. After photo equilibrium (Stage III, Fig. 4e), the hole quasi-Fermi level in TiO_2_ is not positive enough for water oxidation, but the electron quasi-Fermi level can reduce oxygen. Therefore, a steady photocathodic current for oxygen reduction is observed at −0.3 V_RHE_ (Fig. S24) [37]. In addition to TiO_2_, the abnormal photocathodic current is also observed on other n-type semiconductor photoanodes, such as CdS [38] and BiVO_4_ [39]. When the light is off, the stored holes in the OFL are released to TiO_2_ through the RFL (Stage IV, Fig. 4e). Therefore, unlike the OFL, the RFL serves as electron transfer mediator and contributes to the steady photocathodic current in a photoanode.

Besides being found on a semiconductor, an intrinsic faradaic layer is also found on the surface of a metal and a faradaic material. The typical CV curves of Pt and MnO_x_ are shown in Fig. S25. Different from the bipolarized intrinsic faradaic layer on a semiconductor under illumination, the Pt and MnO_x_ indicate only a mono-polarized faradaic layer, either the RFL or OFL, at a given potential. Therefore, intrinsic faradaic layers widely exist at the interfaces of semiconductor/liquid, metal/liquid and faradaic material/liquid, which form faradaic junctions [36,40,41]. According to the above discussion, the bipolarity or mono-polarity of the surface faradaic layer on a semiconductor depends on the applied potential in photoelectrocatalysis. For an n-type semiconductor, if the applied potential is more negative than the positive limit of the potential window of the RFL, and the corresponding quasi-Fermi level of holes is more positive than the negative limit of the potential window of the OFL, the bipolarized surface can be observed, in which photo-generated electrons and holes both transfer to the surface and react with the redox couples in the electrolyte (Fig. 4d). In this case, the photo-induced charge transfer path in photoelectrocatalysis is similar to that in photocatalysis. In contrast, if the applied potential is more positive than the positive limit of the potential window of the RFL, or the corresponding quasi-Fermi level of holes is more negative than the negative limit of the potential window of the OFL, the mono-polarized surface can be observed in photoelectrocatalysis, which is more like electrocatalysis than photocatalysis (Fig. S23).

Though electrical double layer and surface state mechanisms have been proposed to understand the solid/liquid interface in previous studies, the faradaic junction mechanism is different from the two classic mechanisms (Fig. S26). In the electrical double layer mechanism, a Helmholtz layer and a diffuse layer are included in the liquid side, while only electrons accumulate at the solid side without real thickness [42]. The concept of surface states was first proposed by Tamm in 1932 and derives from dangling bonds on the solid surface, referred to as intrinsic surface states. In the 1960s, extrinsic surface states, such as surface adsorption, were also proposed to understand semiconductor/liquid or semiconductor/metal interfaces [43–46]. However, like the electrical double layer mechanism, the two kinds of surface states also indicate no real thickness on the solid side and only electron transfer is involved. In contrast, a faradaic layer is a self-existent substance with real thickness from the bulk, and the thickness changes with different potentials. Coupled ion and electron transfer is also experimentally proven at the TiO_2_/electrolyte interface for the first time in this study. Moreover, the potential window of an intrinsic faradaic layer can be located out of the band gap, while the positions of surface states are usually located within the band gap of a semiconductor (Fig. 2c and d). Therefore, the faradaic junction mechanism is an amendment of the electrical double layer and surface state mechanisms.

Since the charge transfer process at the solid/liquid interface is complex, great efforts have been made to determine the surface structure of the solid in working conditions via different *in situ* characterization methods. However, the surface of a solid is usually amorphous and the structure and composition depend on light, electricity, temperatures and chemical surroundings, which could change in a very short time (<μs) [47]. Moreover, proton transfer is usually involved. Therefore, it is a great challenge to elucidate the exact structure and composition of the surface of a solid in working conditions. The faradaic junction mechanism suggests that the exact structure and composition of a solid surface changes quickly with the applied potential (Fig. S27). Therefore, electrochemical potential space can be introduced as a simplified descriptor for the structure and composition of the solid surface, in which it is not necessary to know the exact structure and composition of the surface. It is just like momentum (k) space is introduced to describe electronic states in solid physics though exact positions of electrons also cannot be measured in a solid. Moreover, theoretical calculations have been widely used to further understand the charge transfer process at the solid/liquid interface in previous studies [48–50]. However, those theoretical studies can only describe defects and molecular adsorption on the rigid surface, and are not suitable for calculating the coupled electron and ion transfer process on the dynamic surface in this study. In future work, we will try to develop new calculation methods to understand the special charge transfer process in a solid/liquid faradaic junction.

## CONCLUSION

For the first time, we find and prove a bipolarity characteristic of the intrinsic faradaic layer on the TiO_2_ semiconductor surface under illumination by *in situ* XPS, *in situ* EPR and TOF-SIMS methods. The composition of the RFL and OFL are Ti^+4^O_2−x_(OH)_2x_ + H^+^ + e^−^ ↔ Ti^+3^O_1−x_(OH)_2x+1_ and Ti^+4^O_2−x_(OH)_2x_ + H_2_O + h^+^ ↔ Ti^+4^O^−1^O_1−x_(OH)_2x+1_ + H^+^, respectively. Meanwhile, the potential windows of the RFL and OFL are obtained as −0.4 ∼ 0.5 V_RHE_ and 1.2 ∼ 2.0 V_RHE_ via the electrochemical method. In photocatalysis, the RFL and OFL both serve as electron and hole transfer mediators, while the bipolarity or mono-polarity of the intrinsic faradaic layer on a semiconductor depends on the applied potential in photoelectrocatalysis. The new characteristic of bipolarity can be used to explain abnormal photocathodic current in a photoanode. The faradaic junction mechanism can offer a universal and clear description for charge transfer at the solid/liquid interface, which is a bridge between photochemistry, electrochemistry and photoelectrochemistry.

## Supplementary Material

nwac249_Supplemental_File
