# Supplementary material for: Bipolarized intrinsic faradaic layer on a semiconductor surface under illumination
Source: Natl Sci Rev. 2022 Nov 4;10(4):nwac249. doi: 10.1093/nsr/nwac249 (PMC10148736; doi:10.1093/nsr/nwac249)
Supplement: nwac249_Supplemental_File [file nwac249_supplemental_file.pdf]

Supplementary Information

Bipolarized Intrinsic Faradaic Layer on Semiconductor Surface under Illumination

Mengfan Xue[1], Zhiqiang Chu[1], Dongjian Jiang[2], Hongzheng Dong[2], Pin Wang[1], Gengzhi Sun[3], Yingfang Yao[2], Wenjun Luo[2*], Zhigang Zou[1,2]

[1]Eco-materials and Renewable Energy Research Center (ERERC), Jiangsu Key Laboratory for Nano Technology, National Laboratory of Solid State Microstructures and Department of Physics, Nanjing University, Nanjing 210093, China

[2]National Laboratory of Solid State Microstructures, College of Engineering and Applied Sciences, Nanjing University, Nanjing 210093, China

[3]Key Laboratory of Flexible Electronics (KLOFE) & Institute of Advanced Materials (IAM), Nanjing Tech University, Nanjing 211816, China

*Email: wjluo@nju.edu.cn;

Methods

Preparation of TiO_2 and Fe_2O_3 films

A TiO_2 film was deposited on a FTO substrate by a hydrothermal method [1]. The precursor solution was obtained by dissolving 15 mL HCl (36%-38% by weight) into 15 mL deionized water, while adding 0.45 mL titanium butoxide by stirring. The precursor solution was transferred into a 25 mL telfon steel autoclave. The FTO glass was immersed into the precursor solution as a substrate. The hydrothermal process was kept at 150 °C for 9 hours. After cooling down, the deposited film was washed and calcined in a muffle furnace at 450 °C for 1 hour in air.

A Ti doped hematite film was also deposited on a FTO substrate by hydrothermal method [2]. The precursor solution was obtained by adding 7.5 mmol $FeCl_3 \cdot 6H_2O$ and 50 μL $TiCl_3$ into 100 mL deionized water, and adjusting the pH of the solution with 0.6 mL HCl (36%-38%). The solution was then transferred into a 100 mL telfon steel autoclave and a FTO glass was used as a substrate. The hydrothermal process was kept at 100 °C for 4 hours. The deposited film was washed and then calcined in a muffle furnace at 675 °C for 15 min in air and taken out at 575 °C.

Pretreatment of single crystal TiO_2

A commercial single crystal rutile (110) TiO_2 (Hefei, Kejing) was calcined at 700℃ for 200 min in the mixture carrier gas with 10% H_2 and 90% Ar to improve its conductivity for photoelectrochemical measurement [3]. The back side was connected to a copper rod with molten indium to form ohmic contact. Then the back side of single crystal TiO_2 electrode was sealed with insulting silica glue.

Characterization of samples

The crystal structure of the samples was characterized by X-ray diffraction (XRD, smartlab, 9 kW). The morphology of the samples was investigated by scanning electron microscope (SEM, Gemini 500) with an accelerating voltage of 10 kV and transmission electron microscope (TEM, Tecnai F20). The X-ray photoelectron spectroscopy (XPS, Thermo Scientific XPS K-alpha) were performed with an Al Kα X-ray source. The binding energy of the C1s peak at 284.6 eV was used to calibrate the XPS data. Ions depth profiles in the samples were obtained by time-of-flight secondary-ion mass spectroscopy (TOF-SIMS, Ion tof Gmhb 5) with a detection mode of negative ions. A beam of 30 keV Bi^+ was used as primary ions, with an analyzing area of 91 * 91 μm^2. The Cs^+ ions with 1 keV were used to sputter the samples was performed in an area of 250 * 250 μm^2.

In situ X-ray photoelectron spectroscopy (XPS, Thermofisher Escalab 250Xi) was performed in the dark and under a Xe lamp illumination for 30 min. To prepare the TiO_2 sample with Mn^{2+} and Ag^+ as electron and hole imaging agents, the solution of 5 mM CH_3COOAg and 10 mM $(CH_3COO)_2Mn$ was dropped onto the surface of TiO_2 and dried in the dark. In situ electron paramagnetic resonance (EPR, Bruker E500) was performed in the dark and under a Xe lamp illumination for 30 min in N_2.

Isotope labeling experiments

The isotope labeling experiments on TiO_2 were performed in the mixture solution of D_2O and $H_2{}^{18}O$ (volume ratio of D_2O/ $H_2{}^{18}O$, 1:1) in the dark and under a Xe lamp illumination for 2 h. In order to make D diffuse more deeply into TiO_2, methanol was added into the mixture solution (volume ratio of methanol/mixed water, 1:5) as a hole scavenger. Moreover, an electrochemically reduced TiO_2 was carried out at -0.4 V_{RHE} for 1 h in 1 M phosphate buffer aqueous solution with D_2O as solvent.

Photo-deposition of MnO_x and Ag

A TiO_2 thin film was immersed into the aqueous solution with 5 mM CH3COOAg or 10 mM $(CH3COO)_2Mn$ or both, respectively, and then they were irradiated by a Xe

lamp for 5 min, 10 min and 30 s, respectively. Moreover, the co-deposition on single crystal TiO_2 was carried out in the aqueous solution with 5 mM CH3COOAg and 10 mM $(CH3COO)_2Mn$ under illumination for 2 min.

(Photo-)electrochemical measurements

The (photo-)electrochemical measurement of films was investigated in a three-electrode cell by using an electrochemical workstation (Shanghai Chenhua, CHI 760e) under illumination. The light source was an AM 1.5G sunlight simulator (Beijing, China Education Au-light) with the light intensity of 100 mW/cm^2. The prepared films were used as the working electrodes. An Ag/AgCl electrode and a graphite rod were used as a reference electrode and a counter electrode, respectively. The electrolyte was 1 M phosphate buffer solution with pH~7. All of the potentials were calculated at reversible hydrogen electrode (RHE) scale following the formula: $V_{RHE} = V_{Ag/AgCl} + 0.059*pH + 0.197$.

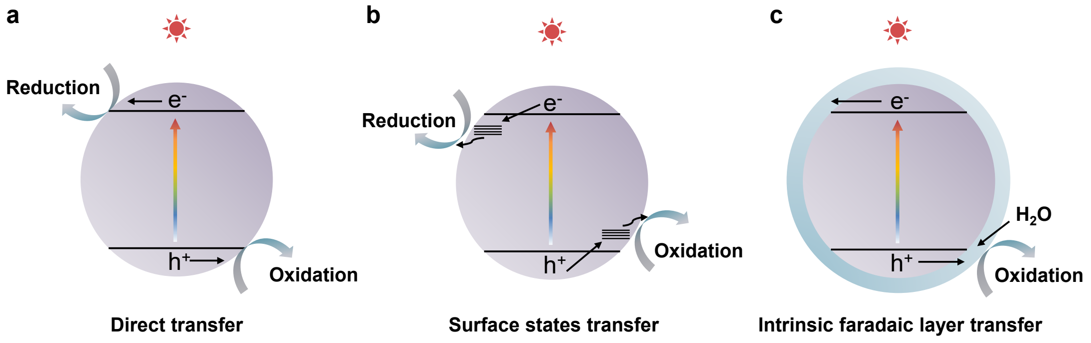

Figure S1. Different interface charge transfer models, direct transfer (a), surface states transfer (b) and intrinsic faradaic layer transfer (c).

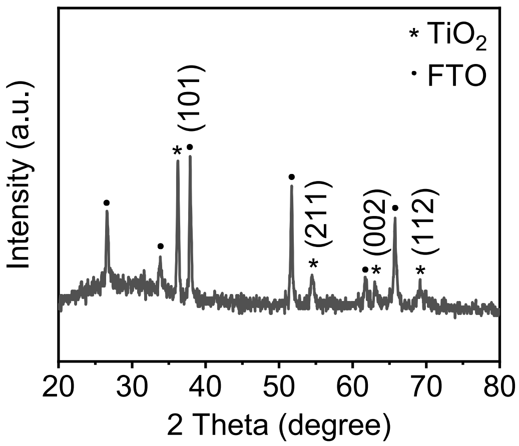

Figure S2. X-ray diffraction (XRD) pattern of rutile TiO_2 (JCPDS 21-1276).

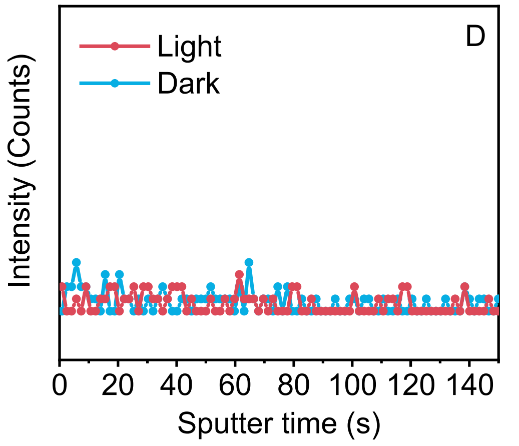

Figure S3. TOF-SIMS spectra of D depth profile of TiO_2 in the dark and under illumination, in the mixture solution of D_2O and $H_2{}^{18}O$.

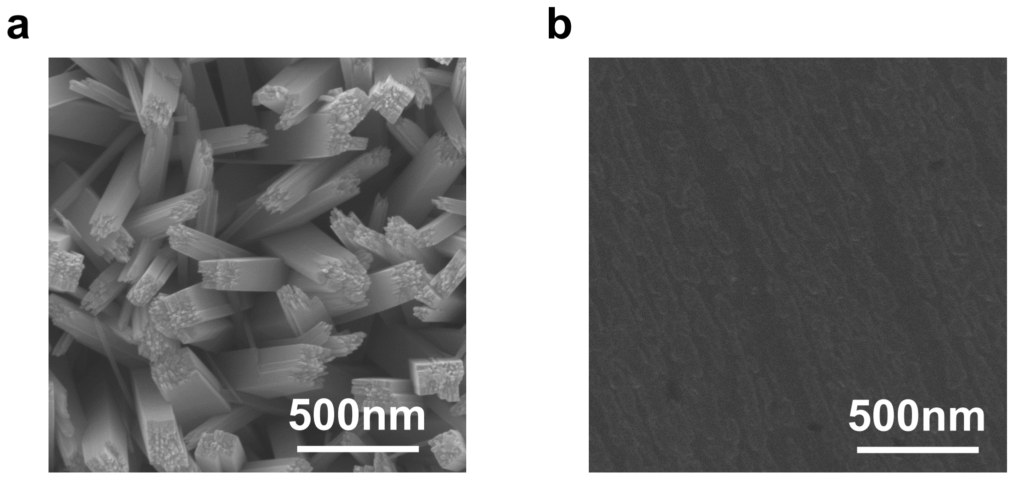

Figure S4. Surface SEM images of TiO_2 nanorod (a) and single crystal TiO_2 (110) (b).

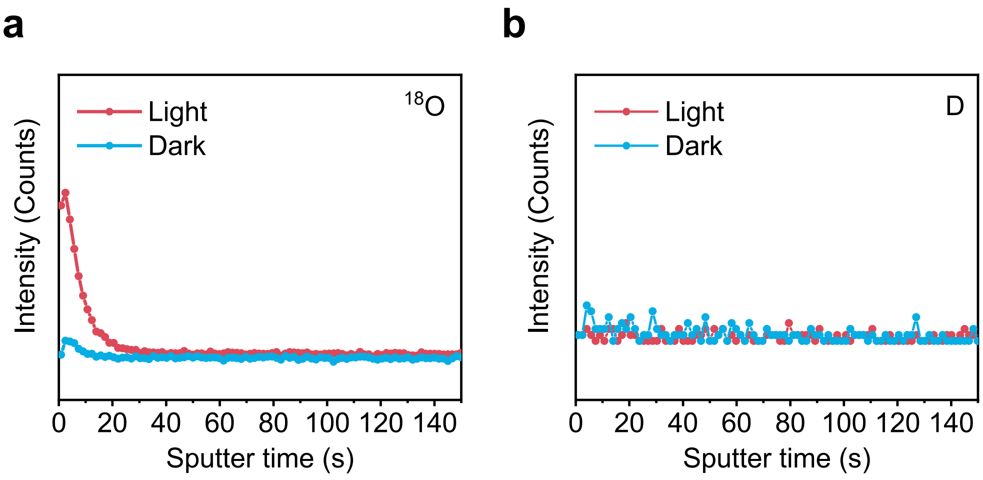

Figure S5. TOF-SIMS spectra of ^{18}O depth profile (a) and D depth profile (b) of single crystal TiO_2 (110) in the dark and under illumination.

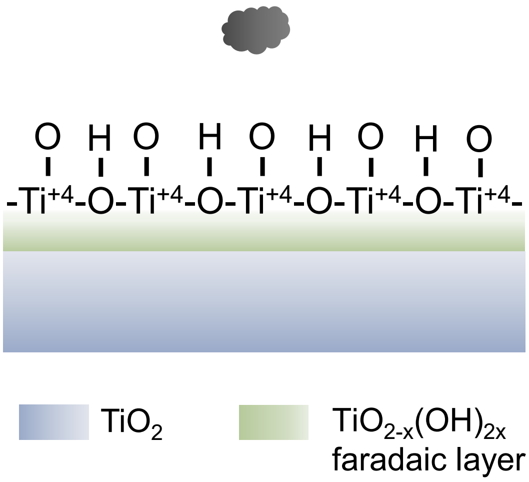

Figure S6. Schematic diagram of surface composition of TiO_2 in the dark.

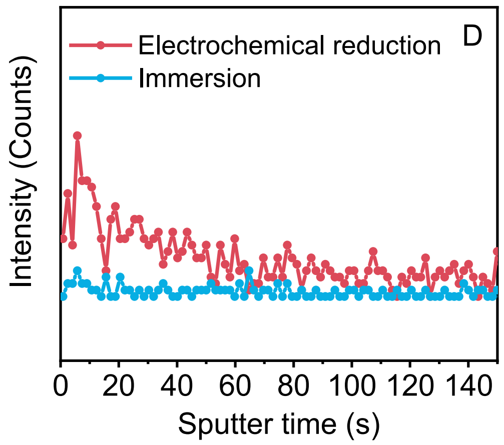

Figure S7. TOF-SIMS spectra of D depth profile of electrochemically reduced TiO_2 at -0.4V_{RHE} in 1M phosphate buffer solution (pH~7) with D_2O as solvent for 1 h, TiO_2 immersed in the mixture solution of D_2O and $H_2{}^{18}O$ in the dark as a reference.

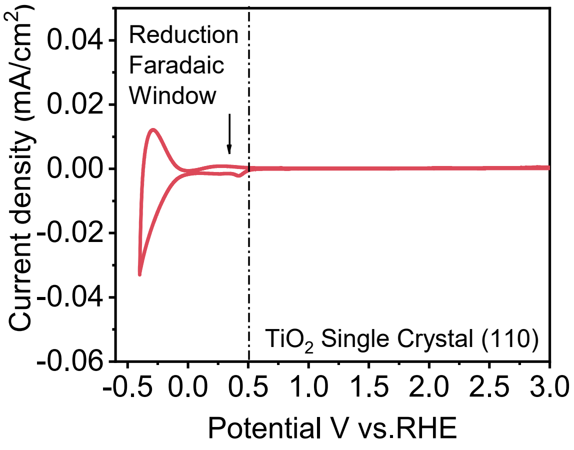

Figure S8. CV curve of TiO_2 single crystal (110) in 1M phosphate buffer solution (pH~7) in the dark.

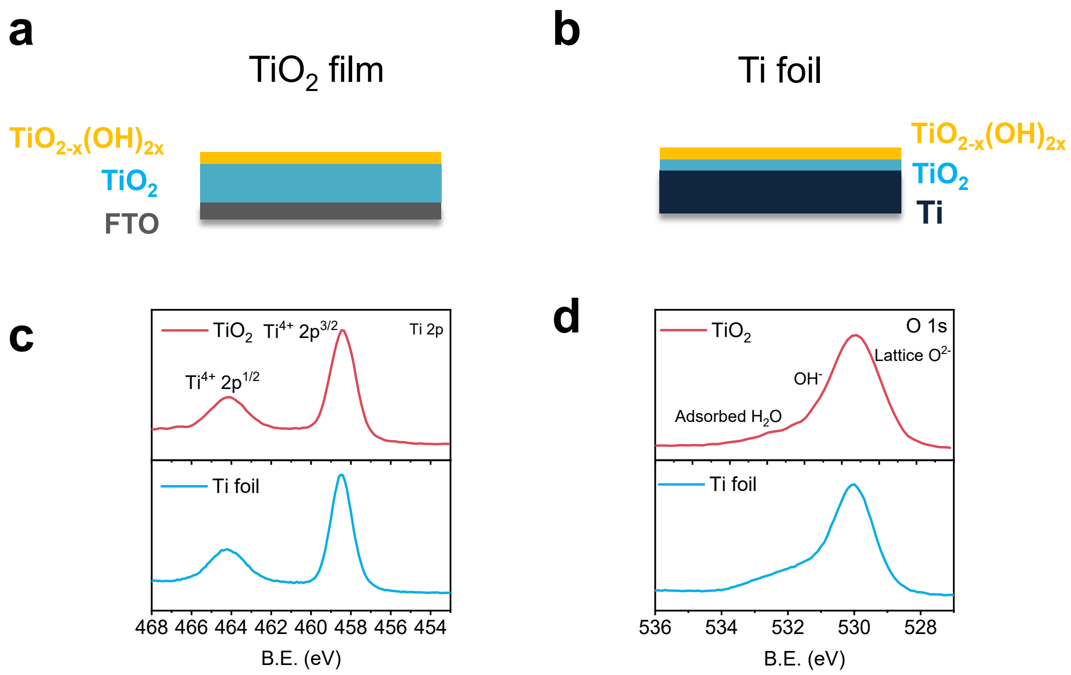

Figure S9. Cross section schematic diagram of TiO_2 (a) and Ti foil (b); XPS spectra for TiO_2 and Ti foil, Ti 2p (c) and O 1s (d).

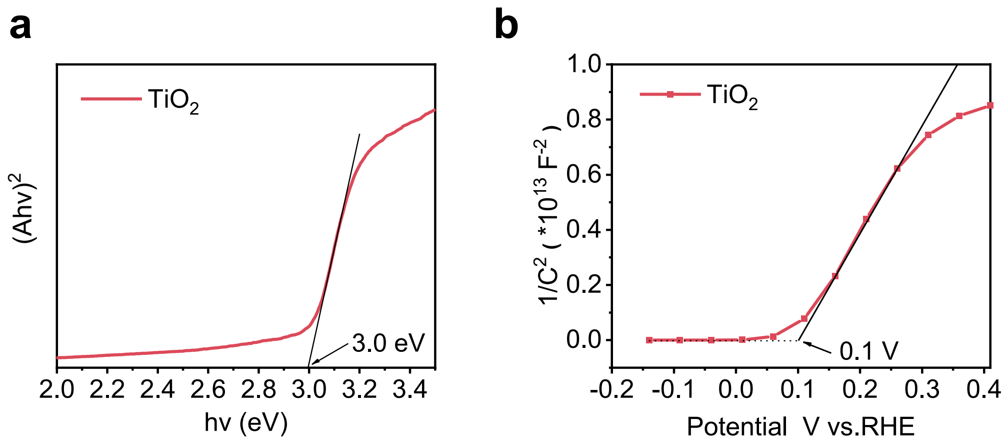

Figure S10. Bandgap (a) and Mott-Schottky plots (b) of TiO_2 in 1M phosphate buffer solution (pH~7).

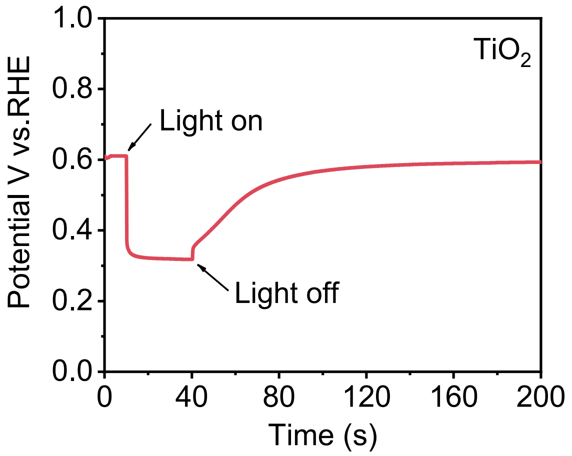

Figure S11. Open-circuit potential (OCP) of TiO_2 in 1M phosphate buffer solution (pH~7) in the dark and under illumination.

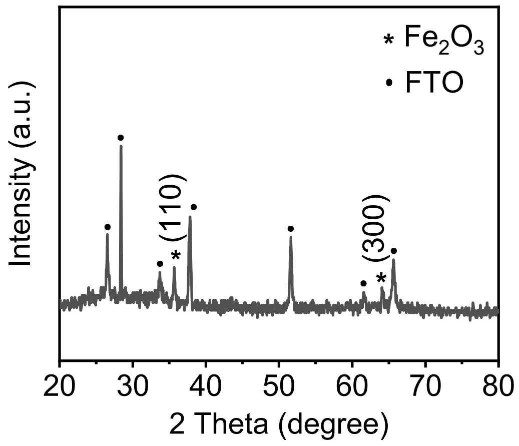

Figure S12. XRD pattern of hematite Fe_2O_3 (JCPDS 33-0664).

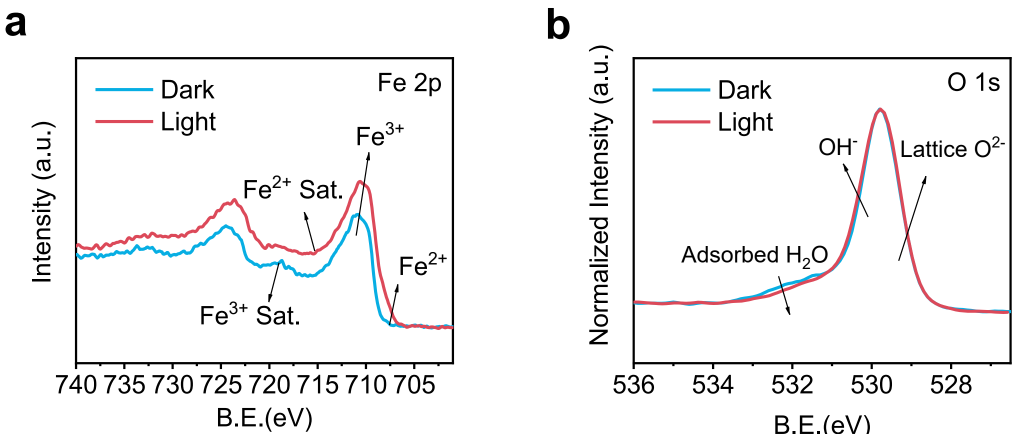

Figure S13. In situ XPS spectra of Fe_2O_3 in the dark and under illumination, Fe 2p (a), O 1s (b).

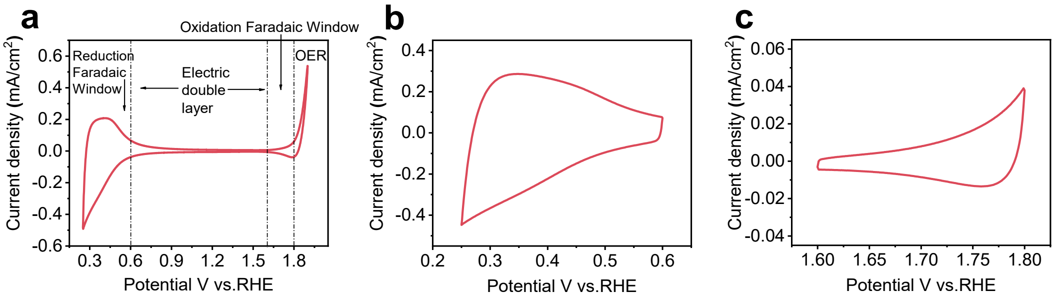

Figure S14. CV curve of Fe_2O_3 in 1M phosphate buffer solution (pH~7) in the dark, 100 mV/s, full range (a), reduction faradaic layer (b) and oxidation faradaic layer (c).

Table S1. Faradaic reactions and potential window of bipolarized intrinsic faradaic layer on Fe_2O_3.

**Reduction Faradaic layer**	**Potential Window**
$Fe^{+3}O_x(OH)_{3-2x} + H^+ + e^- \leftrightarrow Fe^{+2}O_{x-1}(OH)_{4-2x}$	0.25 - 0.6 V
**Oxidation Faradaic layer**	**Potential Window**
$Fe^{+3}O_x(OH)_{3-2x} + h^+ + OH^- \leftrightarrow Fe^{+4}O_x(OH)_{4-2x} + H_2O$	1.6 - 1.8 V

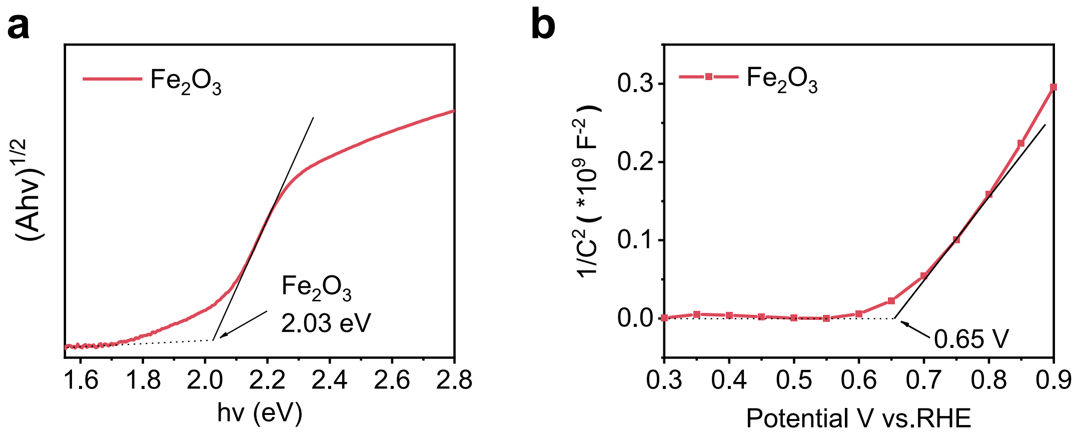

Figure S15. Bandgap (a) and Mott-Schottky plots (b) of Fe_2O_3 in 1M phosphate buffer solution (pH~7).

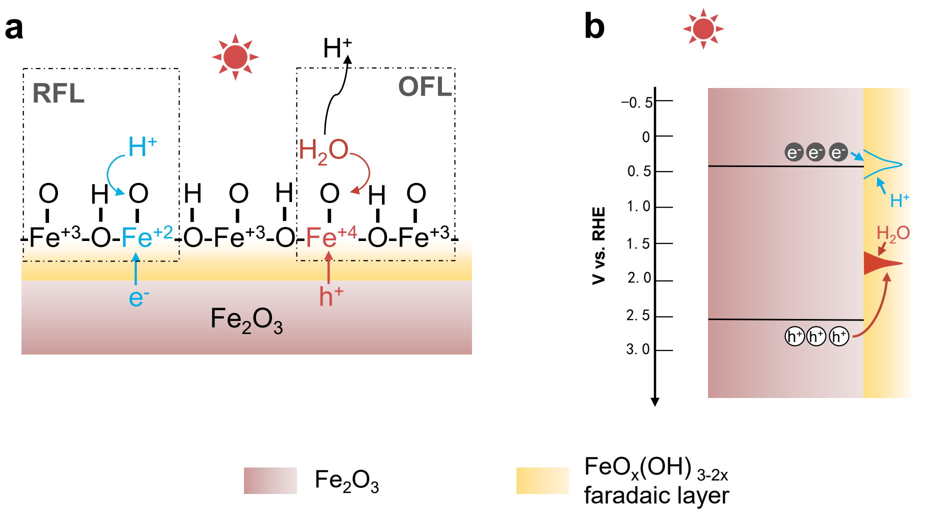

Figure S16. Schematic diagram for Fe_2O_3 in photocatalytic process under illumination, surface composition (a) and energy band positions and potential windows of RFL and OFL (b).

Similar bipolarized intrinsic faradaic layer is also observed on hematite Fe_2O_3 surface (Figure S14). New peaks of Fe^{2+} reduced from Fe^{3+} and decrease of H_2O content are appeared under illumination compared with in the dark [4] (Figure S13). Therefore, Fe^{2+} is the RFL of Fe_2O_3. Since detection of Fe^{4+} on Fe_2O_3 is already shown by FT-IR under photoelectrochemical measurement in previous study, we suggest that Fe^{4+} as the oxidation product of Fe_2O_3 [5]. The reactions of reduction and oxidation faradaic layer and the corresponding potential window for Fe_2O_3 are shown in Table S1.

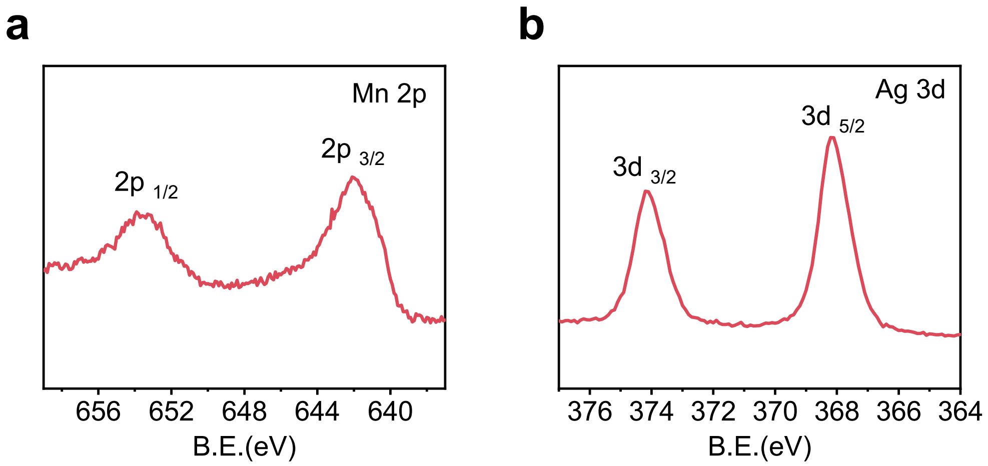

Figure S17. XPS spectra of TiO_2 with imaging agents Mn^{2+} and Ag^{+}, Mn 2p (a) and Ag 3d (b).

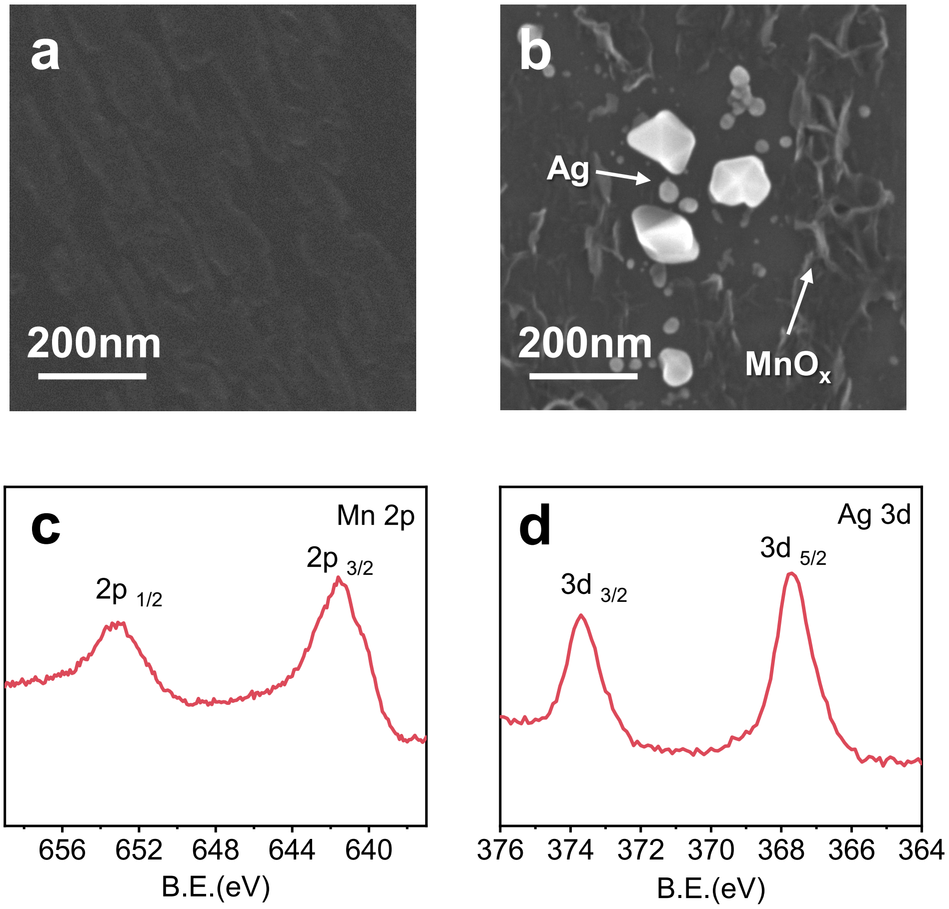

Figure S18. SEM images of single crystal TiO_2 (110) without imaging agents (a) and TiO_2 (110) with imaging agents Mn^{2+} and Ag^{+} (b); XPS spectra of TiO_2 (110) with imaging agents Mn^{2+} and Ag^{+}, Mn 2p (c) and Ag 3d (d).

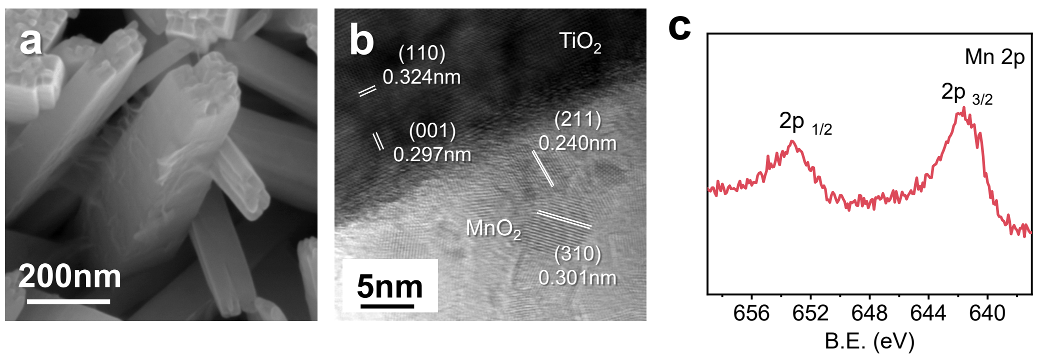

Figure S19. SEM (a), TEM (b) and XPS spectra of Mn 2p (c) of TiO_2 with single photo-deposition of MnO_x,

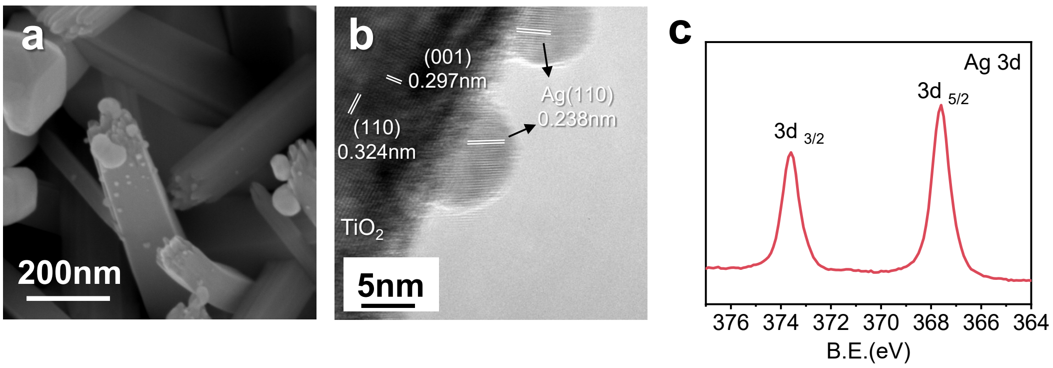

Figure S20. SEM (a), TEM (b) and XPS spectra of Ag 3d (c) of TiO_2 with single photo-deposition of metal Ag.

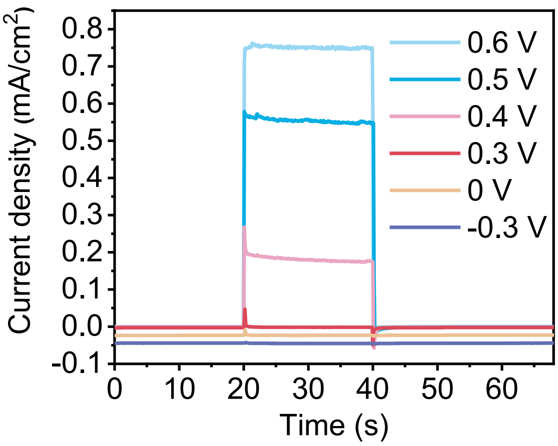

Figure S21. I-t curves of TiO_2 in 1M phosphate buffer solution (pH~7) at different potentials under chopped illumination.

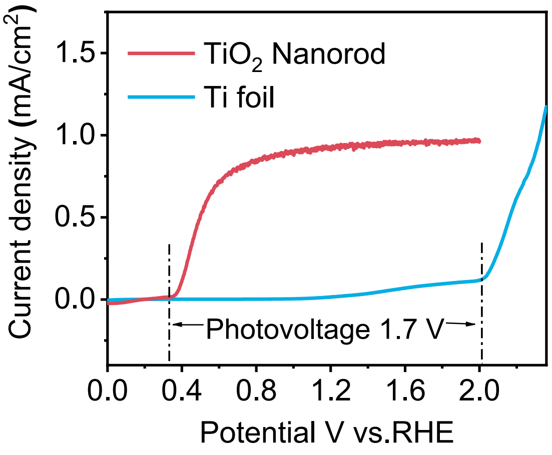

Figure S22. LSV curves of TiO_2 and Ti foil in 1M phosphate buffer solution (pH~7),

20 mV/s. The onset potential for TiO_2 is about 0.3 V under illumination, and for Ti foil is about 2.0 V in the dark. In a Faradaic junction, the photovoltage means the difference between the quasi-fermi levels of electrons and holes, which can be obtained by the difference between a photo-onset potential of TiO_2 and a dark onset potential of Ti foil [6]. Therefore, the photovoltage in TiO_2 is about 1.7 V.

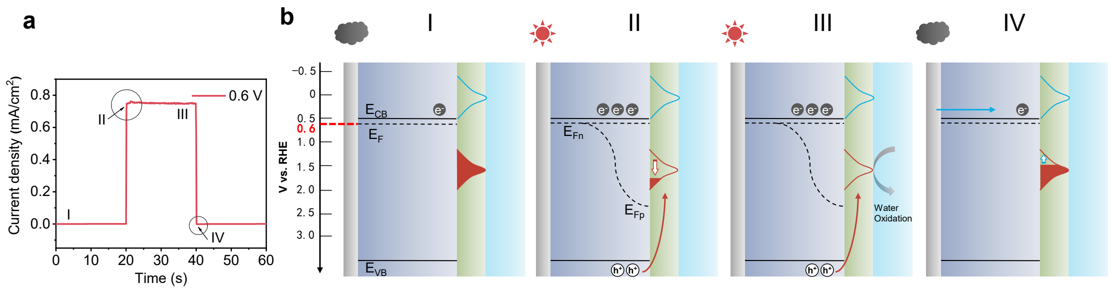

Figure S23. (a) I-t curves of TiO_2 at 0.6 V $_{RHE}$, (b) schematic diagrams for energy band positions and potential windows of RFL and OFL of TiO_2 for charge transfer processes at 0.6 V_{RHE} in the dark and under illumination. E_{VB} and E_{CB} are the valence band and conduction band of a semiconductor, respectively; E_F represents the Fermi level of electrons; E_{Fn} and E_{Fp} represent the quasi-Fermi levels of electron and hole, respectively.

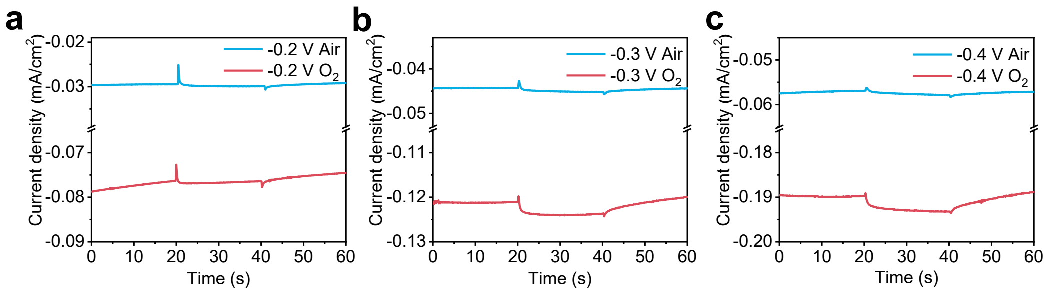

Figure S24. I-t curves of TiO_2 at -0.2 V $_{RHE}$ (a), -0.3 V $_{RHE}$ (b) and -0.4 V $_{RHE}$ (c) in 1M phosphate buffer solution (pH~7) in air and with O_2 bubbling under chopped illumination.

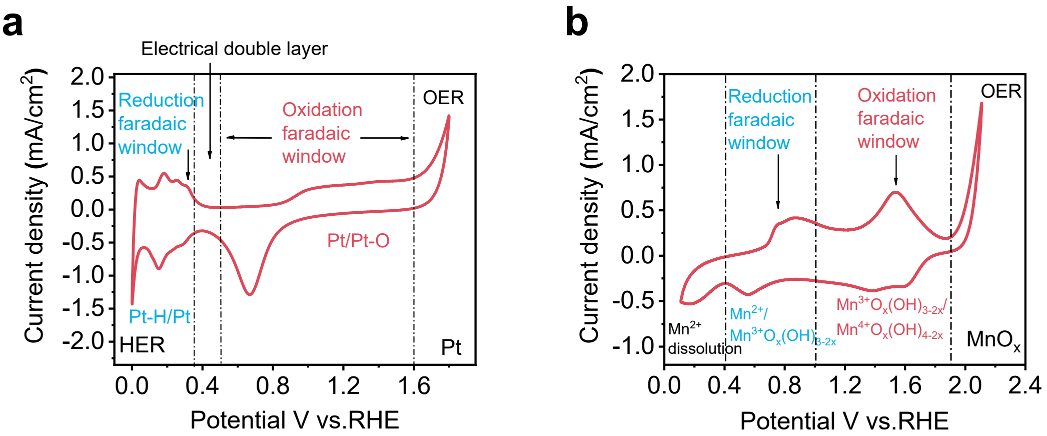

Figure S25. CV curves of Pt (a) and MnO_x (b) in 1M phosphate buffer solution (pH~7), 100 mV/s.

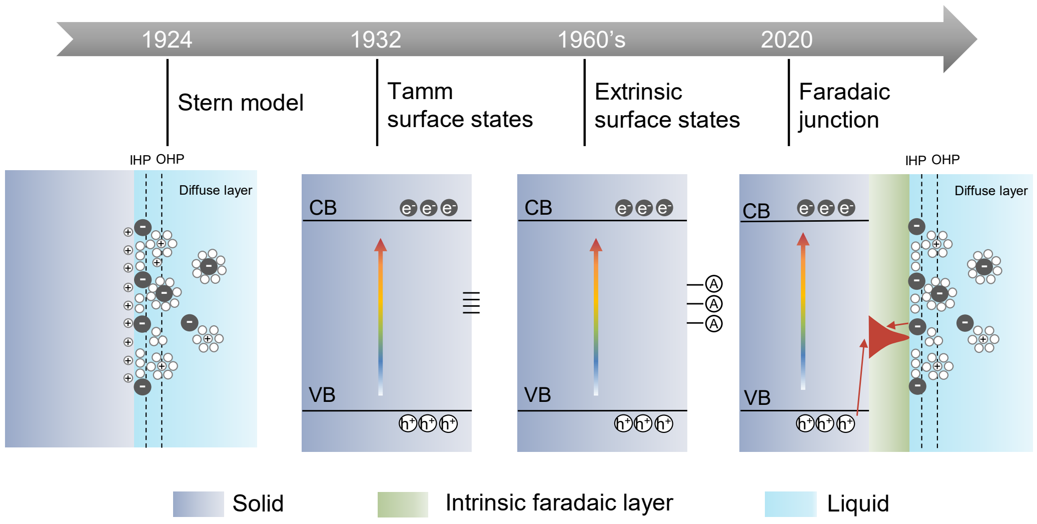

Figure S26. Timeline for the development of different mechanisms on solid surface/interface.

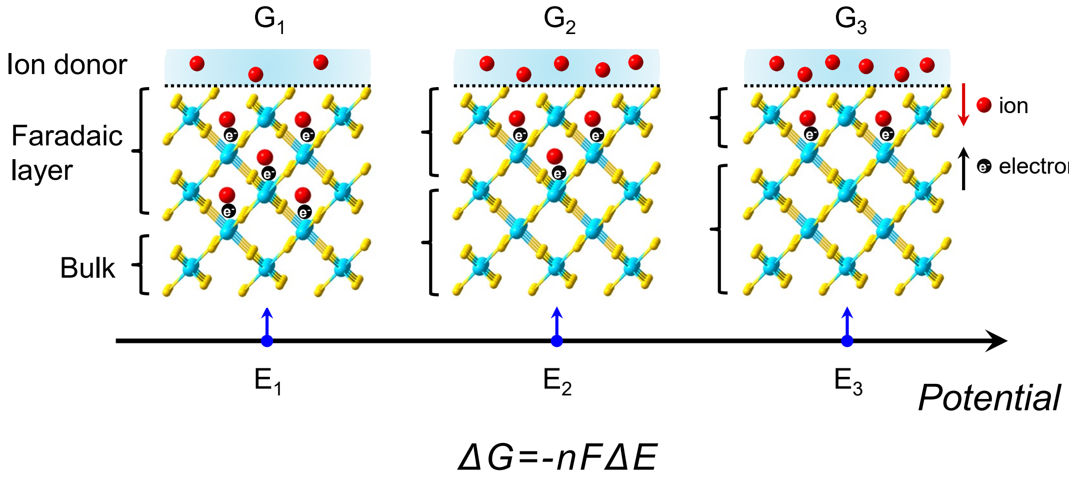

Figure S27. The corresponding relationship between the structure and composition vs. the potential of a faradaic layer. The potential difference can be calculated from the formula of $\Delta G=-nF\Delta E$ (ΔG is the difference between the Gibbs free energy of the faradaic layer and a reference electrode, n is the electron number of the reaction, F is the Faraday constant).

References

1. Xia X, Luo J, Zeng Z, et al. Integrated photoelectrochemical energy storage: solar hydrogen generation and supercapacitor. *Sci Rep* 2012; **2**: 981.
2. Wang T, Luo W, Wen X, et al. Nonequilibrium Ti^{4+} Doping Significantly Enhances the Performance of Fe_2O_3 Photoanodes by Quenching. *ChemNanoMat* 2016; **2**: 652-5.
3. Yang Q, Zhu H, Hou Y, et al. Surface polaron states on single-crystal rutile TiO_2 nanorod arrays enhancing charge separation and transfer. *Dalton Trans* 2020; **49**: 15054-60.
4. Ling Y, Wang G, Reddy J, Wang C, et al. The Influence of Oxygen Content on the Thermal Activation of Hematite Nanowires. *Angew Chem Int Ed* 2012; **124**: 4150-5.
5. Zandi O, Hamann TW. Determination of photoelectrochemical water oxidation intermediates on haematite electrode surfaces using operando infrared spectroscopy. *Nat Chem* 2016; **8** :778-83.

6. Chen X, Zhu K, Wang P, et al. Reversible Charge Transfer and Adjustable Potential Window in Semiconductor/Faradaic Layer/Liquid Junctions. *iScience.* 2020; **23**: 100949.
